# Design of Low-Cost and High-Strength Titanium Alloys Using Pseudo-Spinodal Mechanism through Diffusion Couple Technology and CALPHAD

**DOI:** 10.3390/ma14112910

**Published:** 2021-05-28

**Authors:** Chaoyi Ding, Chun Liu, Ligang Zhang, Di Wu, Libin Liu

**Affiliations:** 1 School of Material Science and Engineering, Central South University, Changsha 410083, China; 12010024@hnist.edu.cn (C.D.); ligangzhang@csu.edu.cn (L.Z.); 2 School of Mechanical Engineering, Hunan Institute of Science and Technology, Yueyang 414006, China; 3 State Key Laboratory of Advanced Technology for Materials Synthesis and Processing, Wuhan University of Technology, Wuhan 430070, China; cliu@whut.edu.cn

**Keywords:** low-cost titanium alloy, pseudo-spinodal mechanism, CALPHAD, diffusion couple, 3D strength model

## Abstract

The high cost of development and raw materials have been obstacles to the widespread use of titanium alloys. In the present study, the high-throughput experimental method of diffusion couple combined with CALPHAD calculation was used to design and prepare the low-cost and high-strength Ti-Al-Cr system titanium alloy. The results showed that ultra-fine α phase was obtained in Ti-6Al-10.9Cr alloy designed through the pseudo-spinodal mechanism, and it has a high yield strength of 1437 ± 7 MPa. Furthermore, application of the 3D strength model of Ti-6Al-xCr alloy showed that the strength of the alloy depended on the volume fraction and thickness of the α phase. The large number of α/β interfaces produced by ultra-fine α phase greatly improved the strength of the alloy but limited its ductility. Thus, we have demonstrated that the pseudo-spinodal mechanism combined with high-throughput diffusion couple technology and CALPHAD was an efficient method to design low-cost and high-strength titanium alloys.

## 1. Introduction

Titanium alloy is an important strategic metal material with low density, high specific strength, and excellent corrosion resistance [1,2,3]. Such alloys, sometimes referred to as “air metal”, “marine metal”, or “smart metal”, are used in many applications in the aerospace, marine, chemical, and medical fields [4,5,6,7,8,9,10,11,12].

However, titanium alloys are much more expensive compared with steels and other metallic materials, which limits their applications. The development, production, and characterization of low-cost titanium alloys are current research hotspots [13,14,15,16,17]. For these reasons, cheap alloying elements, such as Al, Fe, and Cr, instead of expensive ones, such as V, Mo, and Nb, are used to design titanium alloys and achieve a cut in cost [18,19,20,21,22,23,24]. A variety of low-cost titanium alloys have been developed, such as Timetal 62S (Ti-6Al-1.7Fe-0.1Si), RMI VM (Ti-6.4Al-1.2Fe), KS Ti-531C (Ti-4.5Al-2.5Cr-1.2Fe-0.1C), TIX-90 (Ti-1.5Fe-0.5O-0.05N), SAT-64AW (Ti-6Al-4V-10Cr-1.3C), Ti-35421 (Ti-3Al-5Mo-4Cr-2Zr-1Fe), and Ti8LC (Ti-6Al-1Mo-1Fe). However, the strength of these alloys is less than 1100 MPa, which limits their applications in some specific fields, such as aircraft and military equipment, where the strength is a sensitive consideration. Thus, there is a scope for developing Ti alloys that are both low cost and high strength.

The precipitation of acicular secondary α phase in the β matrix is the primary reason for the high strength of titanium alloys [25,26]. Nag et al. [27] proposed a novel pseudo-spinodal mechanism of the precipitation of α phase in the β matrix. It was indicated that when the composition of the alloy is located near the intersection of the Gibbs free-energy curves of the α and β phases, the variation of free energy between the α and β phases provides a driving force for direct transformation of β to α phase, which leads to the precipitation of α phase in a matrix of β phase within a short time.

The trial-and-error method that, traditionally, is used in the design of alloys is time consuming, laborious, and expensive. In contrast, the CALPHAD method can be used to calculate phase composition, and fraction, driving force of phase transition and other characteristics affecting alloy properties in a short amount of time. It can provide effective guidance for alloy design, hence, reducing the number of experiments performed. Additionally, the alloy samples with various constituents can be prepared in a timely way by utilizing high-throughput diffusion couple technology, which can further decrease the number of experiments performed [28].

In the present work, the low-cost and high-strength Ti-Al-Cr system alloys were designed using high-throughput diffusion couple technology and CALPHAD. The influence of Cr content on the microstructure and properties of Ti-6Al-xCr alloys was determined. The Gibbs free energy of the α and β phases in the alloy was calculated by CALPHAD. The Cr content of the alloy that has the ultra-fine α phase was determined using the pseudo-spinodal mechanism.

## 2. Materials and Methods

**Figure 1:**
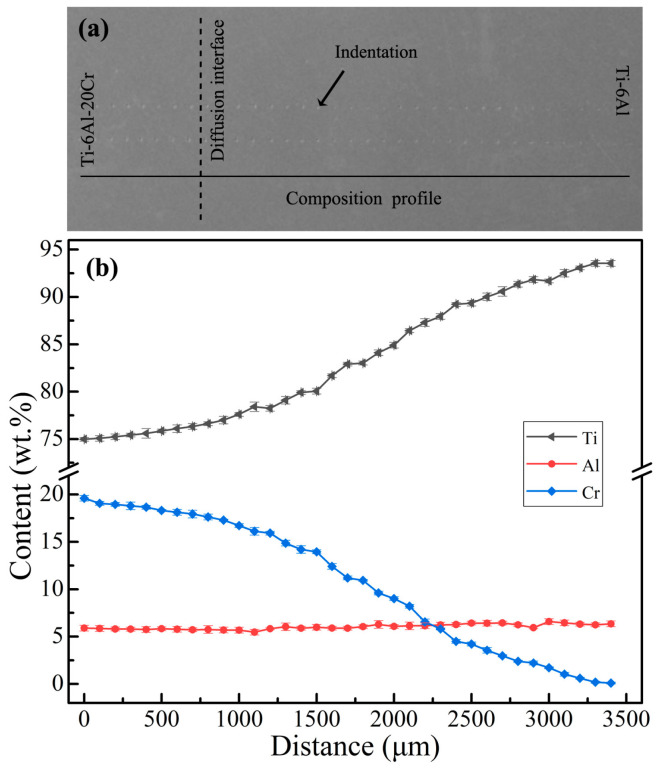
Micro-hardness indentation (**a**) and variation curve of composition with diffusion distance (**b**) of the (Ti-6Al)–(Ti-6Al-20Cr) diffusion couple.

Pure Ti, Al, and Cr (purity = 99.99 wt.%) were used as the starting metals. Ti-6Al and Ti-6Al-20Cr alloys for the preparation of diffusion couple were prepared by smelting the starting metals 5 times in a non-consumable vacuum arc furnace. The aforementioned alloys were wire cut, using electrical discharge machining, into blocks that measured 10 mm × 10 mm × 5.5 mm and 10 mm × 10 mm × 2.5 mm. The two blocks were clamped together, and the interfaces of the fixture and the alloys were covered with tantalum foil. Diffusion welding was conducted in a vacuum furnace at 1000 °C for 6 h. Subsequently, the couple specimen was annealed at 1100 °C for 240 h in a vacuum quartz tube, resulting in a composition gradient. After cooling in air, the diffusion couple specimen was cut in half, with one half being used as a solid solution sample and the other half being aged at 600 °C for 6 h. Finally, the samples were ground and polished for characterization. Vickers hardness of the samples was measured with a micro-indentation hardness tester (Buehler5104; Buehler, Lakebraw, IL, USA) with a loading scale of 1.961 N for 15 s. Along the diffusion direction of Cr, the distance between successive indentations was 100 μm (Figure 1a). A field emission scanning electron microscope (SEM) (JSM-7001F; JEOL, Akishima, Tokyo, Japan) and an electron probe (JXA–8230; JEOL, Akishima, Tokyo, Japan) were used to characterize the micro-structure and composition at 1–3 μm near an indentation.

The designed Ti-6Al-10.9Cr alloy was prepared by melting the starting metals 5 times in a non-consumable vacuum arc furnace. The β/α transition temperature of the alloy, as determined by metallographic method, was 835 ± 5 °C. The alloy was forged at 1000 °C to ensure that the β grains were fully broken; then, the second forging was carried out at 810 °C. The forged samples were solution treated in the β single-phase region for 0.5 h and aged at 600 °C for 6 h. The phase composition was characterized using X-ray diffraction (XRD) (Ultima III; Rigaku, Rigaku, Akishima, Tokyo, Japan) with Cu-Kα radiation. The aged samples were processed into dog bone specimens for tensile test at room temperature. The micro-morphology of the alloy was analyzed using a transmission electron microscope (TEM) (Tecnai G2 20; FEI, Hillsboro, OR, USA). The tensile performed tests were using a universal materials testing machine (MTS 810; MTS Systems, Minneapolis, MN, USA) at a cross-head displacement rate of 0.8 mm/min. The fracture morphologies of the stretched tensile specimens were investigated using a field emission scanning electron microscope (SEM) (JSM–7001F JEOL, Akishima, Tokyo, Japan). The Gibbs free energy, the phase fraction, and the phase composition of the α and β phases were calculated using the Thermo-Calc software.

## 3. Results

### 3.1. Composition Design of Ti-6Al-xCr Alloys

The composition distribution of the (Ti-6Al)–(Ti-6Al-20Cr) diffusion couple is displayed in Figure 1b. It was seen that, with the increase in diffusion distance, the Cr diffused into the matrix alloy from the left side, the Ti content increases, the Cr content decreased, and the Al content was practically unchanged.

**Figure 2:**
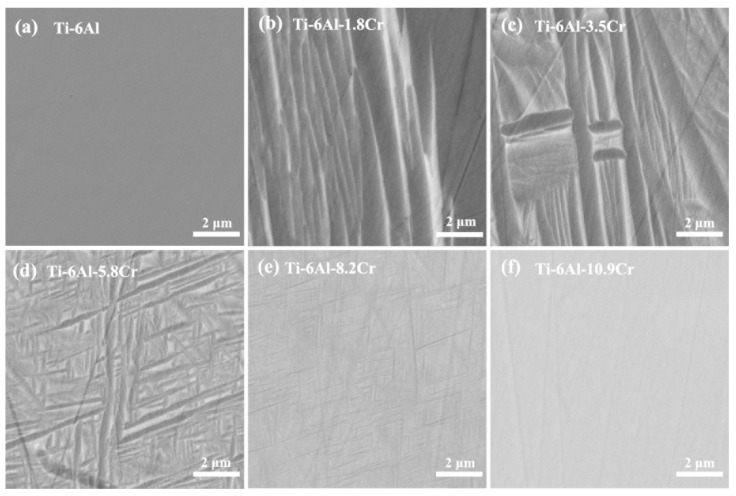
SEM, backscattered electron images of Ti-6Al-xCr alloys, taken from (Ti-6Al)–(Ti-6Al-20Cr) diffusion couple, after quenching at 1100 °C.

Figure 2 shows the micro-structure of the Ti-6Al-xCr alloys quenched from the β single-phase region. When the Cr content was 0 wt.%, the alloy was composed only of α phase, and the coarse lath-shaped martensitic α′ was formed at a Cr content of 1.8 wt.%. The micro-structure of the α′ phase is gradually refined with increasing Cr content. When the Cr content was increased to 8.2 wt.%, many thin needle-like martensitic α′ were interweaved with each other in the β matrix. When the Cr content reached 10.9 wt.%, the α′ phase was hardly observed, and the alloy was composed of a single β phase.

**Figure 3:**
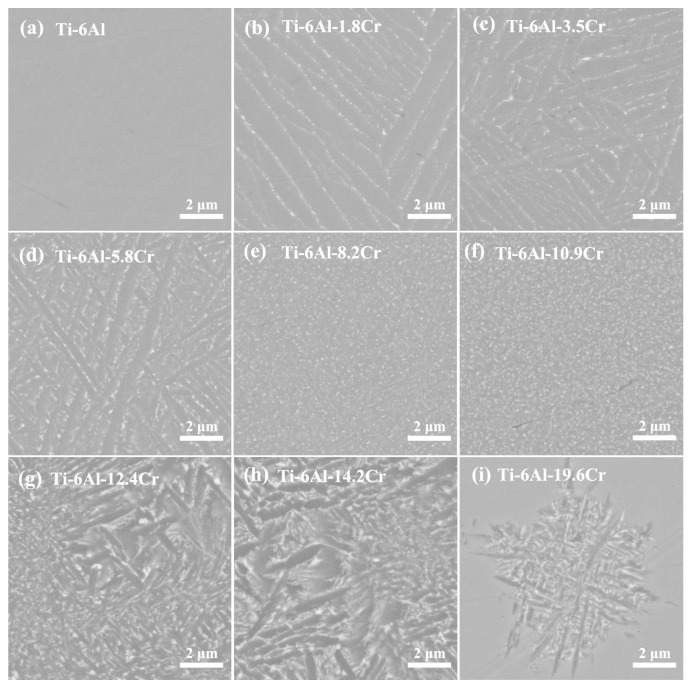
SEM, backscattered electron images of Ti-6Al-xCr alloy taken from the (Ti-6Al)–(Ti-6Al-20Cr) diffusion couple, after aging at 600 °C.

Figure 3 presents the micro-structure of the Ti-6Al-xCr alloys quenched from the β phase region and aged at 600 °C for 6 h. When the Cr content was 0 wt.%, the alloy consisted only the α phase. When the Cr content was increased to 1.8 wt.%, the coarse lath-like α phase was obtained. The volume fraction of the α phase in the alloy gradually decreased with the increase in the Cr content, and the morphology of α phase changed from sheet-like to needle-like and point-like. The α phase of the Ti-6Al-10.9 Cr alloy had the finest structure, with the β matrix being filled with a needle-dot-like α phase, which was very small and, hence, hard to identify. The structure of the α phase began to gradually become coarse again with the further increase in Cr content. When the Cr content increased to 19.6 wt.%, a small amount of dendritic α phase was distributed in the β matrix.

**Figure 4:**
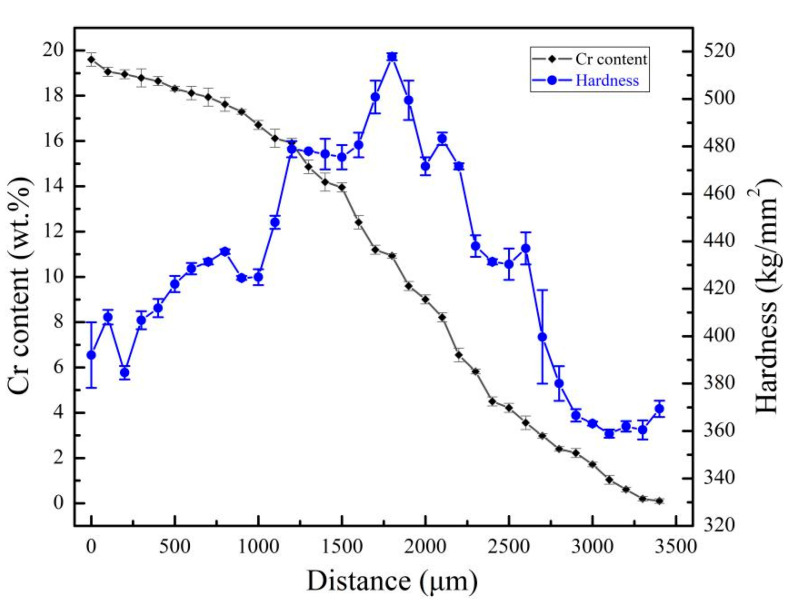
Cr content–hardness relationship of the series Ti-6Al-xCr alloys, after aging at 600 °C.

Figure 4 shows the variation of hardness with Cr content in Ti-6Al-xCr alloys subjected to solution treatment in the β single-phase region and aging at 600 °C. When the Cr content was less than 10.9 wt.%, the hardness increased with the increase in Cr content. When the Cr content was above 10.9 wt.%, the hardness decreased with increasing Cr content. The highest hardness (518 kg/mm^2^) was obtained with a Cr content of 10.9 wt.%. The corresponding micro-structural analysis (Figure 3f) reveals that when the Cr content was 10.9 wt.%, the alloy had a fine and well-dispersed α phase.

**Figure 5:**
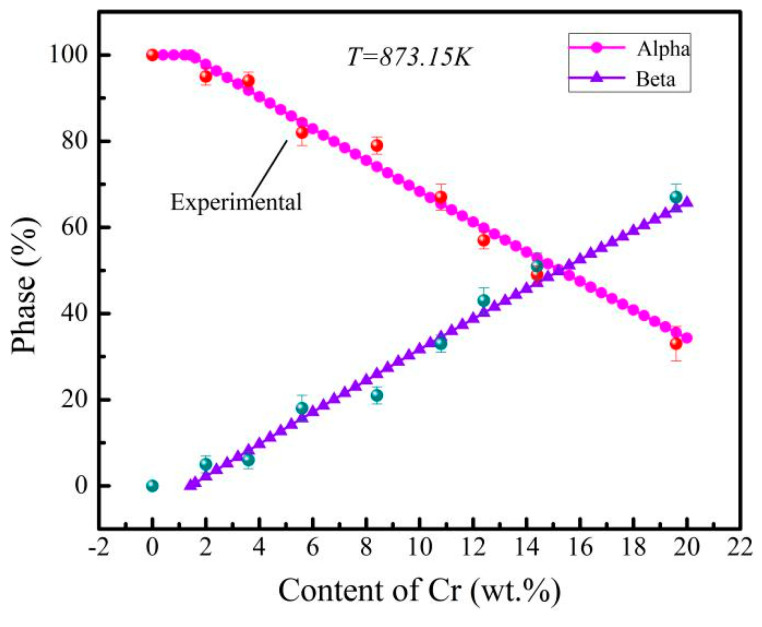
The calculation results of the phase composition of Ti-6Al-xCr alloys, after aging at 600 °C.

The variation of the phase fractions of the α and β phases of the Ti-6Al-xCr alloys, aged at 600 °C, with Cr content, is shown in Figure 5. Note that in this figure, the laves phase is ignored. The calculated results showed that, with increase in Cr content, the α phase decreased while the β phase increased, which was consistent with the experimental statistical results.

**Figure 6:**
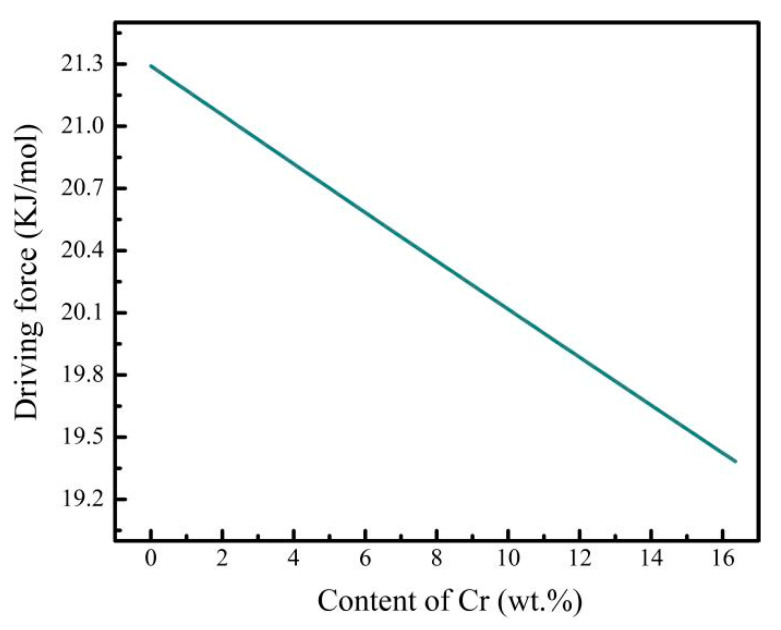
Calculated variation of driving force of α phase precipitation with Cr content.

The calculated driving force of α phase nucleation decreased with the increase in Cr content (Figure 6). When the Cr content was less than 10.9 wt.%, α′ martensite was obtained in Ti-6Al-xCr alloys after quenching, and the morphology of α phase in the aged Ti-6Al-xCr alloy depended on that of α′ martensite. When the Cr content was greater than 10.9 wt.%, the driving force of α phase precipitation continuously decreased with the increase in Cr content. Compared with Ti-6Al-10.9Cr alloy, the number of α phase nucleations in Ti-6Al-12.4Cr alloy decreased markedly, but the difference of nucleation driving force of α phase in the two alloys was very small (Figure 6). Therefore, the change of nucleation rate of the α phase cannot be explained by a change of driving force. A detailed explanation of this phenomenon is provided in the discussion section.

### 3.2. Micro-Structure and Mechanical Properties of Ti-6Al-10.9Cr Alloy

**Figure 7:**
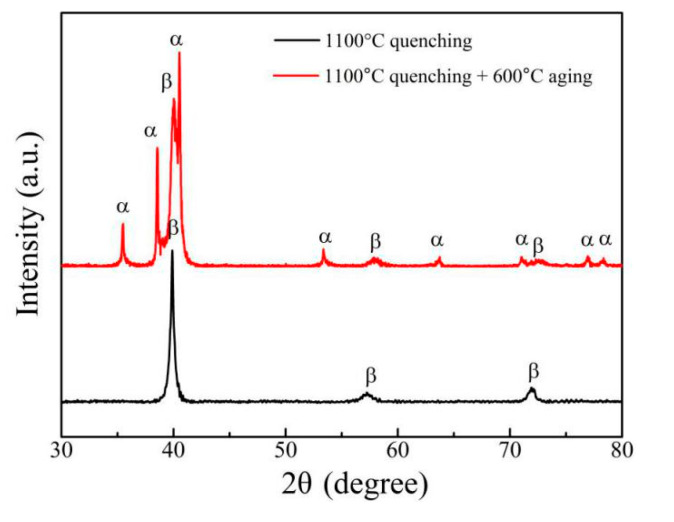
XRD patterns of the quenched and aged Ti-6Al-10.9Cr alloy.

Figure 7 displays the XRD patterns of the quenched and aged Ti-6Al-10.9Cr alloy. Only the β phase could be detected in the alloy, after quenching at 1100 °C. This was mainly because that as a β phase stabilizing element, high-content Cr could hinder the martensitic transformation during the quenching process. The α phase became the predominant phase, after further aging in (α + β) region (600 °C), implying continuous conversion from the β to α phase. 

**Figure 8:**
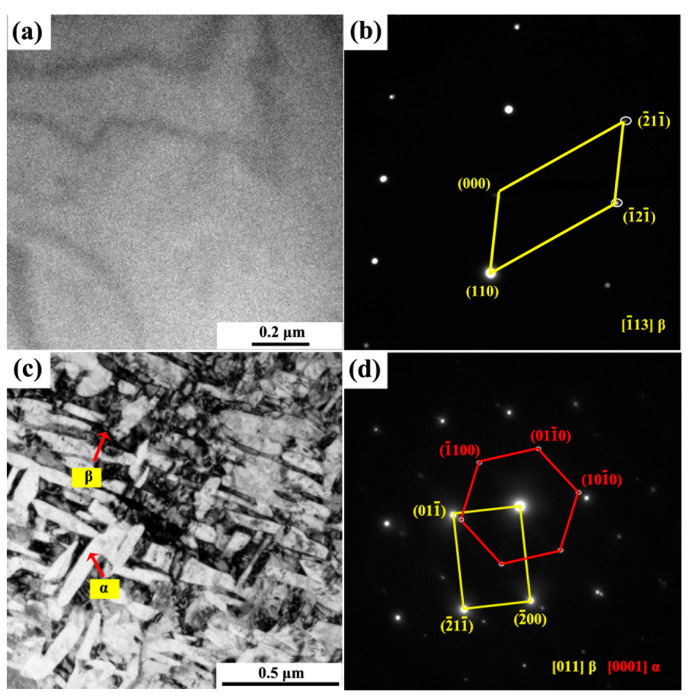
TEM images (**a**,**c**) and electron diffraction(**b**,**d**) of Ti-6Al-10.9Cr alloy: (**a**,**b**) after quenching at 1100 °C, (**c**,**d**) after aging at 600 °C.

Figure 8 shows the TEM characterization results of the quenched (Figure 8a,b) and aged Ti-6Al-10.9Cr alloy (Figure 8c,d).The bright field image (Figure 8a) of the quenched alloy and the selected electron diffraction (Figure 8b) in direction of [$\bar{1}13$]β demonstrated that the alloy was composed of the β phase, with no martensitic α′ or ω phase being found. As shown in Figure 8c, a large amount of cross-distributed α phase was presented in the β matrix. The α phase in the aged alloy had a length of~ 300 nm and a width of ~30 nm. The selected area electron diffraction (Figure 8d) in the [011]β and [0001]α directions further proved that the alloy consisted of α and β phases, which agreed well with the XRD analysis.

**Figure 9:**
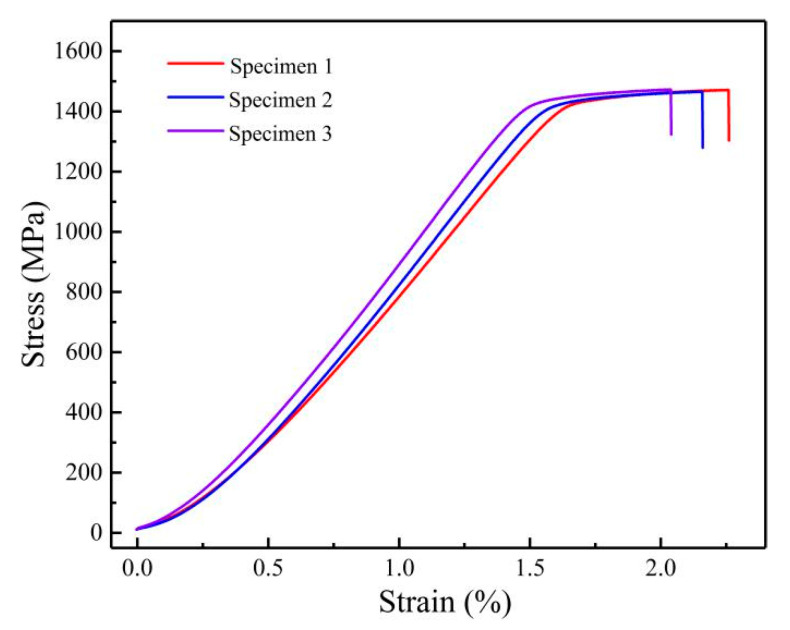
Tensile test results: forged Ti-6Al-10.9Cr alloy in β single-phase solution treatment plus aging at 600 °C.

The tensile modulus, tensile yield strength, ultimate tensile strength, tensile elongation at fracture of the alloy, which was given solid solution treatment in β single-phase region and aged at 600 °C were 108 ± 4 GPa, 1437 ± 7 MPa, 1465 ± 9 MPa, and 2.1 ± 0.1%, respectively (Figure 9).

**Figure 10:**
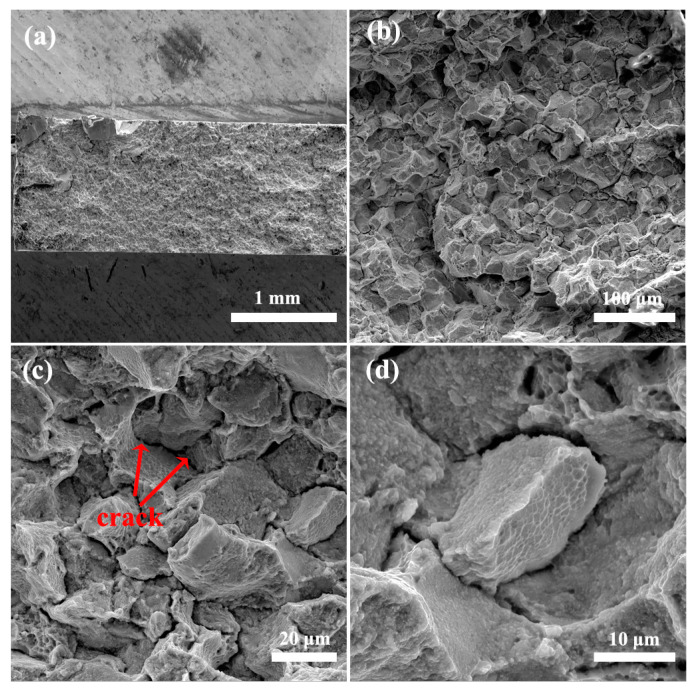
Fracture morphology of the forged Ti-6Al-10.9Cr alloy after tensile deformation: (**a**) overview microstructure, (**b**–**d**) SEM images at different magnifications.

The fracture surface of a tensile specimen at the end of the test was relatively flat (Figure 10). Many tiny cracks (as marked by the red arrow in the Figure 10c) extended along the β grain boundary. Only a few small dimples could be observed on the fracture surface.

## 4. Discussion

### 4.1. Pseudo-Spinodal Mechanism

The main reason for the high strength of Ti-6Al-10.9Cr alloy is the ultra-fine precipitation of ultra-fine secondary α phase in the β matrix. Arguments to support this postulate are now detailed. 

**Figure 11:**
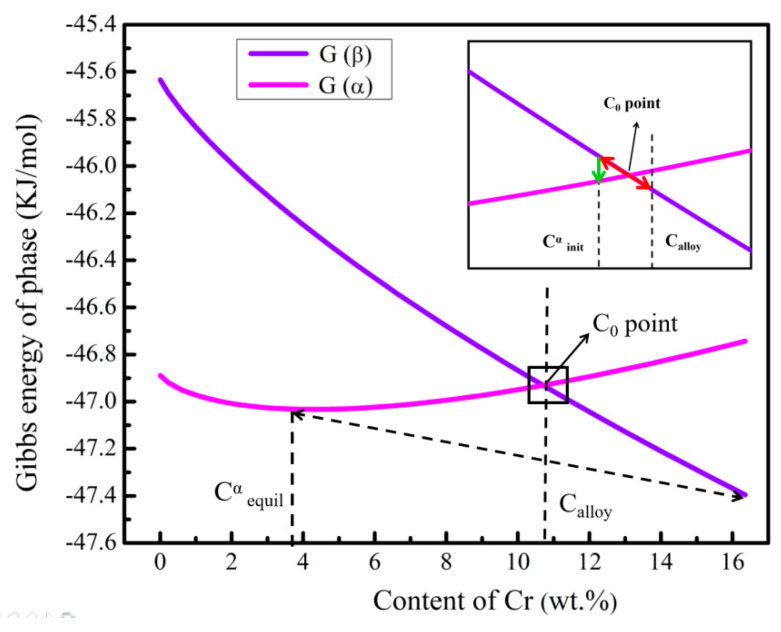
Calculated Gibbs energy curves of α and β phases in Ti-6Al-10.9Cr alloy, at 600 °C.

As mentioned previously, the nucleation rate of the α phase continuously decreased when Cr content was higher than 10.9 wt.%. However, the nucleation number of α phase in Ti-6Al-12.4Cr alloy decreased significantly compared with the case for Ti-6Al-10.9Cr alloy. The Gibbs free-energy curves of α and β phases of Ti-6Al-xCr alloy at 600 °C were calculated (Figure 11) to find out the reason for the anomaly [29,30,31]. It was seen that the intersection of the α and β phases free-energy curves(C_0_ point) was located at the Cr content of 10.7 wt.%, which is very close to 10.9 wt.%.

It has been indicated that during aging treatment, the pseudo-spinodal mechanism would work to precipitate the ultra-fine α phase, when the alloy composition is very close to the intersection of the free energy curves of α and β phases [32,33,34,35]. As shown in Figure 11, when the initial composition of the alloy (C_alloy_) was close to the C_0_ point, the slight composition fluctuation of the alloy (red arrow) causes the composition of the alloy to fluctuate from the right side of the C_0_ point (the free energy of the β phase is lower than that of the α phase) to the left (the free energy of the β phase is higher than that of the α phase). The significant difference in the free energies of the α and β phases provided the driving force for the nucleation of the α phase (green arrow). When the alloy composition was far from the C_0_ point, a slight fluctuation of the composition is not enough to cause the change of free energy in the alloy. Therefore, the precipitation nucleation rate of α phase would be greatly reduced at the alloy compositions far away from the C_0_ point. The theory could well explain why a very high nucleation rate was obtained, in the absence of heterogeneous nucleation. The Cr content in the alloy was far from the C_0_ point with the further increase in Cr content. The precipitation of the α phase began to follow the traditional nucleation-growth mechanism. Meanwhile, the nucleation rate of the α phase was much lower than that consistent with the pseudo-spinodal mechanism. 

### 4.2. Strengthening Mechanism of the Aged Ti-6Al-xCr Alloys

The Cr content had a marked influence on the micro-structure of the alloy, specifically, the thickness and volume fraction of the α phase, and the strength of the alloy depended on the micro-structure. Three reasons are presented to explain this finding. First, the main strengthening mechanism of titanium alloys is α/β interface strengthening [36]. The results given Figure 3 and Figure 4 clearly showed that the thickness and volume fraction of the α phase are important factors that affect the strength of the alloy. Second, the α phase had a precipitation strengthening effect, because the strength of the α phase, which has hcp structure, is considered to be higher than that of the β matrix, which has bcc structure [37,38,39,40]. Third, Cr is an important solution strengthening element in titanium alloys. The increase in Cr content results in the change of α and β phase compositions in the alloy. Therefore, the solution strengthening effect of Cr needs to be considered.

The strength of the multi-component and multi-phase plastic materials conforms to the equivalent strain theory [41,42]. Combined with the Hall–Petch formula, the yield strength ($\sigma_{M}$) of a multi-phase alloy can be expressed by:

(1) $$\sigma_{M}=f_{A}\left(\sigma_{0,A}+m_{A}l_{A}^{-\frac{1}{2}}\right)+f_{B}\left(\sigma_{0,B}+m_{B}l_{B}^{-\frac{1}{2}}\right)+f_{C}\left(\sigma_{0,C}+m_{C}l_{C}^{-\frac{1}{2}}\right)$$

where $f_{i}$ *(i = A, B, C…)* is the volume fraction of each phase (*f_A_ + f_B_ + f_C_ +* … = 1), $\sigma_{0,\;i}$ (*i = A, B, C…*) is the lattice friction coefficient of each phase (which depends on the structure and composition of each of the phases); *m*_i_ is a coefficient that depends on the composition and structure of each phase; and *l*_i_ is the half-thickness or spacing of each of the phases. Therefore, the yield strength of a the two-phase Ti alloy can be described as follows:

(2) $$\sigma_{M}=f_{\alpha }\left(\sigma_{0,\alpha }+m_{\alpha }l_{\alpha }^{-\frac{1}{2}}\right)+f_{\beta }\left(\sigma_{0,\beta }+m_{\beta }l_{\beta }^{-\frac{1}{2}}\right)$$

where $f_{\alpha }$ and $f_{\beta }$ are the volume fractions of the α and β phases, respectively; $\sigma_{0,\;\alpha }$ and $\sigma_{0,\;\beta }$ are the lattice friction coefficients of the α and β phases, respectively; $l_{\alpha }$ is half of the thickness of the α phase; and $l_{\beta }$ is half of the equivalent thickness of the β phase. The Cr content within the α and β phases in Ti-6Al-xCr alloy was calculated. The result showed that the Cr content within the α phase is almost unchanged but there are small changes in the β phase. Therefore, $\sigma_{0,\;\alpha }$ and $\sigma_{0,\;\beta }$ in Equation (2) can be set as constants.

According to the quantitative stereo theory in metallographic measurement, L_L_ = V_V_ = A_A,_ (L_L_, V_V_, and A_A_ are the length, area, and volume ratios, respectively). Combined with the equivalent layer spacing formula for a two-phase mesh material matrix [43], the equivalent thickness of β phase (*d_β_*) can be expressed by the relationship:

(3) $$d_{\beta }=\frac{1.258\sqrt{f_{\beta }}}{1-\sqrt{f_{\beta }}}h$$

where *h* is the thickness of the α phase, $l_{\alpha }$ = *h*/2, and $l_{\beta }$ *=* $d_{\beta }$/2.

Then, combining Equations (2) and (3) yields:

(4) $$\sigma_{M}=f_{\alpha }\sigma_{0,\alpha }+f_{\beta }\sigma_{0,\beta }+f_{\alpha }{M\prime }_{\alpha }^{}h^{-\frac{1}{2}}+f_{\beta }{M\prime }_{\beta }^{}{\left(\frac{1.258\sqrt{f_{\beta }}}{1-\sqrt{f_{\beta }}}h\right)}^{-\frac{1}{2}}$$

where $M_{\alpha }^{}$ = (1/2)^−1/2^$M_{\alpha }$ and $M_{\beta }^{}$ = (1/2)^−1/2^$M_{\beta }$.

A proportional relationship between the yield strength and hardness of alloy has been proposed [44,45]. In accordance with this relationship, the yield strength of other alloys was also calculated. The volume fractions of the α and β phases *(*$f_{\alpha }$ and $f_{\beta }$) were calculated using Thermo-Calc software, and the thickness of the α phase (*h*) was obtained using results given in Figure 3.

The calculated values of $f_{\alpha }$, $f_{\beta }$, *h*, and yield strength are listed in Table 1. The values of $\sigma_{0,\;\alpha }$, $\sigma_{0,\;\beta }$, $M_{\alpha }^{\prime }$, and $M_{\beta }^{\prime }$ obtained by least-squares method are 891 MPa, 795 MPa, 96 MPa·μm^1/2^, and 189 MPa·μm^1/2^, respectively. Then, Equation (4) can be expressed as follows:

(5) $$\sigma_{M}=891f_{\alpha }+795f_{\beta }+96f_{\alpha }h^{-\frac{1}{2}}+189f_{\beta }{\left(\frac{1.258\sqrt{f_{\beta }}}{1-\sqrt{f_{\beta }}}h\right)}^{-\frac{1}{2}}$$

**Figure 12:**
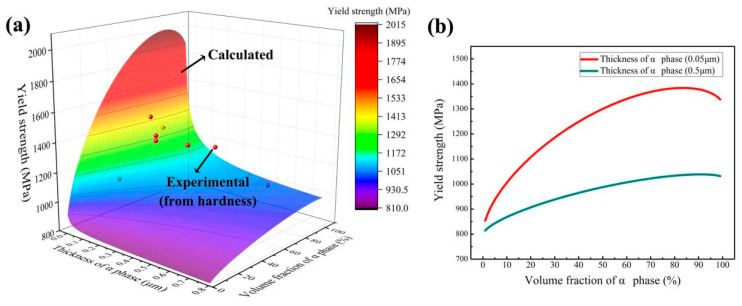
(**a**) Variation of the calculated alloy yield strength with α phase thickness and volume fraction (calculated using Equation (5)), and the experimental results is obtained by proportional conversion based on the proportional relationship between the yield strength and hardness of the existing tensile specimens. (**b**) Variation of yield strength with volume fraction of the α phase for α phase thicknesses of 0.05 and 0.50 μm.

Equation (5) is used to obtain the variation of yield strength with the thickness and volume fraction of the phase, as shown in Figure 12a. The calculated results (note that when $f_{\alpha }$ = 100, the value of *h* cannot be measured) are comparable to the eight sets of experimental data, thereby showing the applicability of the 3D strength model. Therefore, the factors that determined the strength of Ti-6Al-xCr alloy were the thickness and volume fraction of the α phase.

The variation of calculated yield strength with the volume fraction and thickness of the α phase is shown in Figure 12b. When the α phase was thick (0.5 μm), the yield strength of the alloy increased gradually but continuously with increase in α phase volume fraction. This occurs because the strength of the α phase is higher than that of the β phase [46]. The strengthening effect of the α/β interface was less effective than that of the α phase due to the low α/β interface density caused by a coarse α phase. When the α phase was thin (0.05 μm), the yield strength of the alloy increased markedly at first but, then decreased with the increase in α phase volume fraction, with the maximum yield strength (approximately 1350 MPa) being obtained when the α phase volume fraction was approximately 85%. 

### 4.3. Fracture Analysis

The transformation from the α to β phase of the aged Ti-6Al-10.9Cr alloy was consistent with the pseudo-spinodal mechanism, resulting in the precipitation of a large amount of ultra-fine α phase in the β matrix. The analysis of the 3D strength model revealed that the formation of a large amount of α phase with a thickness of ~30 nm (Figure 8c) was the major reason for the high yield strength of the alloy (1437 ± 7 MPa).

A large number of α/β interfaces within the β matrix produced by the ultra-fine α phase could effectively prevent the dislocations from sliding, resulting in the high strength of the β grain. It was difficult for fracture and deformation of the β grain to occur during the tensile process, and the cracks can only spread along the β grain boundary (Figure 10c). Therefore, the main fracture pattern of the alloy was inter-granular fracture. Although many ultra-fine α phases could markedly increase the strength of the alloy, the deformation of the β grain was greatly hindered, resulting in very low ductility (tensile elongation at fracture = 2.1 ± 0.1%).

Compared to the tensile properties of other low-cost and high-strength Ti-based alloys listed in Table 2 [47,48,49,50,51], Ti-6Al-10.9Cr alloy obtained in the present work showed outstanding tensile strength and inferior ductility, which was attributed to the formation of ultra-fine α phase depicted in Figure 3f. The alloy was composed only of the secondary α phase, after solution treating above the β/α transition temperature and aging. No spherical α phase that can improve the plasticity of the alloy was detected. The deformation occurred directly in the secondary α phase and the β matrix, resulting in the brittle tensile fracture. Ti-4Al-2Fe-1Mn alloy also exhibited poor ductility after solution treating in simple β phase region [50]. However, the duplex micro-structure was obtained, and the ductility increased, when the alloy was forged and heat treated in the two-phase region Therefore, it is hopeful that Ti-6Al-10.9Cr alloy with good ductility can be prepared by improving the heat treatment process.

### 4.4. Alloy Comparisons Based on Raw Materials Costs and Properties

In this study, the proposed Ti-6Al-10.9Cr was designed to replace the Ti-6Al-4V alloy. The price of V is about 30 times that of Ti, while the price of Cr is only 0.63 times that of Ti. The relative raw materials costs and properties for the Ti-6Al-10.9Cr and Ti-6Al-4V alloy [52] are listed in Table 3, and alloy raw materials costs are normalized against those of Ti. The data confirmed that Ti-6Al-10.9Cr alloy offered a saving of raw materials cost at 58% compared to the Ti-6Al-4V alloy, and the tensile strength of the designed Ti-6Al-10.9Cr is higher than that of Ti-6Al-4V alloy, while the Ti-6Al-10.9Cr had a poorer ductility. This suggested that substituting the expensive alloying element of V with lower-cost alloying element of Cr was an effective way to reduce the expense of implant materials and maintain high strength.

### 4.5. Study Limitations and Potential Applications

The limitations of this study are worthy of further discussion. In consideration of practical engineering applications, other mechanical properties of Ti-6Al-10.9Cr alloy, such as fracture toughness and fatigue resistance need to be studied further. Furthermore, only simple forging and heat treatments were designed in the present work to minimize the cost of manufacturing and processing of Ti-6Al-10.9Cr alloy component, which also caused the poor ductility of the alloy. It is also valuable to design a more complicated heat treatment process for the alloy to improve the ductility.

The poor ductility (~2.1%) of Ti-6Al-10.9Cr alloy limit the further applications. The alloy is a promising candidate for high-temperature compression, partly due to the low cost and high strength. Moreover, it is possible to enhance the ductility of the alloy through complicating the heat treatment process, and the optimized Ti-6Al-10.9Cr alloy is expected to be used in various applications, such as aerospace, biomedical implants, and chemical industries.

## 5. Conclusions

On the basis of the pseudo-spinodal mechanism, Ti-6Al-xCr alloys with low cost and high strength were designed using high-throughput diffusion couple technology combined with CALPHAD.

The Cr content of a Ti-6Al-xCr alloy has marked effects on its micro-structure and properties. The composition of Ti-6Al-10.9Cr alloy was located near the intersection of the α and β phases free-energy curves, resulting in the precipitation of ultra-fine α phase in a matrix of β phase via pseudo-spinodal transformation and the very high hardness (518 kg/mm^2^).

The 3D strength model of Ti-6Al-xCr alloys indicated that the major factors that determine the strength of the alloys are the thickness and volume fraction of the α phase.

The designed Ti-6Al-10.9Cr alloy has high yield strength (yield strength = 1437 ± 7 MPa) but low ductility (tensile elongation = 2.1 ± 0.1%). This was due to the formation of a large number of α/β interfaces produced by the ultra-fine α phase.

## Figures and Tables

**Table 1:** Summary of the calculated fractions of the α and β phases ($f_{\alpha }$ and $f_{\beta }$) and thickness of the α phase (*h*), experimentally determined values of hardness, experimental yield strength obtained from hardness, calculated yield strength, and yield strength error.

Composition	α-Phase Fraction, $f_{\alpha }$ (%)	β-Phase Fraction, $f_{\beta }$ (%)	Thickness of α Phase, *h* (μm)	Hardness (kg/mm^2^)	Calculated Yield Strength $\sigma_{M}$ (MPa)	Experimental Yield Strength (MPa)	Yield Strength Error (%)
Ti-6Al	100	0	----	369 ± 3.4	----	1024	----
Ti-6Al-1.8Cr	96.31	3.69	0.523 ± 0.017	363 ± 0.9	1033	1007	2.6
Ti-6Al-3.5Cr	91.04	8.96	0.245 ± 0.012	437 ± 6.8	1108	1212	8.6
Ti-6Al-5.8Cr	82.86	17.14	0.131 ± 0.011	438 ± 4.5	1191	1215	2
Ti-6Al-8.2Cr	74.09	25.91	0.038 ± 0.004	483 ± 2.6	1436	1340	7.2
Ti-6Al-10.9Cr	64.77	35.23	0.026 ± 0.005	518 ± 1.5	1499	1437	4.3
Ti-6Al-12.4Cr	59.85	40.15	0.095 ± 0.008	481 ± 5.2	1215	1334	8.9
Ti-6Al-14.2Cr	54.29	45.71	0.135 ± 0.012	477 ± 6.4	1138	1323	14
Ti-6Al-19.6Cr	34.32	65.68	0.057 ± 0.007	392 ± 13.8	1179	1087	8.5

**Table 2:** Comparison of tensile properties of the Ti-6Al-10.9Cr alloy and other low-cost and high-strength Ti-based alloys adapted from literatures [47,48,49,50,51] (PM: powder metallurgy, LPBF: laser powder bed fusion).

Ti-Based Alloy	Tensile Yield Strength (MPa)	Ultimate Tensile Strength (MPa)	Tensile Elongation (%)
Casted Ti-6Al-10.9Cr	1437	1465	2.1
PM Ti-5Al-5Mo-5V-3Cr	1170–1295	1252–1386	7.3–9.0
LPBFed Ti-12Mo-6Zr-2Fe	1026	--	12.7
Casted Ti-4Al–2Fe-1Mn	--	1266	2.2
PM Ti-5Al-3Mo-2Fe	1303	1422	8.5
Casted Ti-4Mo-3Cr-1Fe	870	1092	41

**Table 3:** Comparison of raw materials costs and properties of Ti-6Al-10.9Cr and Ti-6Al-4V alloys.

Alloy	Raw Materials Cost	Properties			
		Elastic Modulus (GPa)	Tensile Yield Strength (MPa)	Ultimate Tensile Strength (MPa)	Tensile Elongation (%)
Ti-6Al-10.9Cr	0.92	108	1437	1465	2.1
Ti-6Al-4V	2.17	124	1123	1242	5.2

## Data Availability

Not applicable.

## References

[1] Froes F.H., Bomberger H.B. (1985). The Beta Titanium Alloys. JOM.

[2] Nyakana S.L., Fanning J.C., Boyer R.R. (2005). Quick Reference Guide for β Titanium Alloys in the 00s. J. Mater. Eng. Perform..

[3] Ghosh A., Sivaprasad S., Bhattacharjee A., Kar S.K. (2013). Microstructure-fracture toughness correlation in an aircraft structural component alloy Ti-5Al-5V-5Mo-3Cr. Mater. Sci. Eng. A.

[4] Ho W.F., Pan C.H., Wu S.C., Hsu H.C. (2009). Mechanical properties and deformation behavior of Ti-5Cr-xFe alloys. J. Alloys Compd..

[5] Li C., Wu X., Chen J.H., Zwaag S. (2011). Influence of α morphology and volume fraction on the stress-induced martensitic transformation in Ti-10V-2Fe-3Al. Mater. Sci. Eng. A.

[6] Okazaki Y., Mori J. (2021). Mechanical Performance of Artificial Hip Stems Manufactured by Hot Forging and Selective Laser Melting Using Biocompatible Ti-15Zr-4Nb Alloy. Materials.

[7] Bhattacharjee A., Bhargava S., Varma V.K., Kamat S.V., Gogia A.K. (2005). Effect of β grain size on stress induced martensitic transformation in β solution treated Ti-10V-2Fe-3Al alloy. Scr. Mater..

[8] Jackson M., Jones N.G., Dye D., Dashwood R.J. (2009). Effect of initial microstructure on plastic flow be-haviour during isothermal forging of Ti-10V-2Fe-3Al. Mater. Sci. Eng. A.

[9] Skubisz P., Packo M., Mordalska K., Skowronek T. (2013). Effect of high strain-rate thermomechanical pro-cessing on microstructure and mechanical properties of Ti-10V-2Fe-3Al Alloy. Adv. Mater. Res..

[10] Furuhara T., Annaka S., Tomio Y., Maki T. (2006). Superelasticity in Ti-10V-2Fe-3Al alloys with nitrogen ad-dition. Mater. Sci. Eng. A.

[11] Zeng W.D., Zhou Y.G., Yu H.Q. (2000). Effect of beta flecks on low-cycle fatigue properties of Ti-10V-2Fe-3Al. J. Mater. Eng. Perform..

[12] Wang Y., Li X., Alexandrov I.V., Ma L., Dong Y., Valiev R.Z., Chang H., Zhang B., Wang Y., Zhou L. (2020). Impact of Equal Channel Angular Pressing on Mechanical Behavior and Corrosion Resistance of Hot-Rolled Ti-2Fe-0.1B Alloy. Materials.

[13] Guo R.P., Sun B.S., Gao B.B. (2008). Low cost manufacturing technology of titanium alloy used in ordnance equipment. Ordnance Mater. Sci. Eng..

[14] Zadra M., Girardini L. (2014). High-performance, low-cost titanium metal matrix composites. Mater. Sci. Eng. A.

[15] Abdelrhman Y., Gepreel M.A.-H., Abdelmoneim A., Kobayashi S. (2016). Compatibility assessment of new V-free low-cost Ti–4.7Mo–4.5Fe alloy for some biomedical applications. Mater. Des..

[16] Bolzoni L., Ruiz-Navas E.M., Gordo E. (2016). Understanding the properties of low-cost iron-containing powder metallurgy titanium alloys. Mater. Des..

[17] Pan D., Liu B., Xu R., Qiu J., Liu C. (2021). Predicting Workability of a Low-Cost Powder Metallurgical Ti–5Al–2Fe–3Mo Alloy Using Constitutive Modeling and Processing Map. Materials.

[18] Sherman A.M., Sommer C.J., Froes F.H. (1997). The use of titanium in production automobiles: Potential and challenges. JOM.

[19] Montgomery J.S., Wells M.G.H., Roopchand B., Ogilvy J.W. (1997). Low-cost titanium armors for combat vehicles. JOM.

[20] Froes F.H., Friedrich H., Kiese J., Bergoint D. (2004). Titanium in the family automobile: The cost challenge. JOM.

[21] Kosaka Y., Fox S.P., Faller K. (2004). Newly developed titanium alloy sheets for the exhaust systems of mo-torcycles and automobiles. JOM.

[22] Sun M., Li D., Guo Y., Wang Y., Dong Y., Dan Z., Chang H. (2020). The Effect of Heat Treatment on the Microstructure and Mechanical Properties of the Novel Low-Cost Ti-3Al-5Mo-4Cr-2Zr-1Fe Alloy. Materials.

[23] Zhou D.Y., Gao H., Guo Y.H., Wang Y., Dong Y.C., Dan Z.H., Chang H. (2020). High-temperature defor-mation behavior and microstructural characterization of Ti-35421 titanium alloy. Materials.

[24] Koike M., Ohkubo C., Fu J.H., Okabe T. (2005). Evaluation of cast Ti-Fe-O-N alloys for dental applications. J. Jpn. I. Met. Mater..

[25] Wu D., Liu L., Zhang L., Wang W., Zhou K. (2020). Tensile deformation mechanism and micro-void nucleation of Ti-55531 alloy with bimodal microstructure. J. Mater. Res. Technol..

[26] Wang Z.Y., Liu L.B., Wu D., Zhang L.G., Wang W.L., Zhou K.C. (2020). α″ phase-assisted nucleation to obtain ultrafine α precipitates for designing high-strength near-β titanium alloys. Trans. Nonferrous Met. Soc. China.

[27] Nag S., Zheng Y., Williams R., Devaraj A., Boyne A., Wang Y., Collins P., Viswanathan G., Tiley J., Muddle B. (2012). Non-classical homogeneous precipitation mediated by compositional fluctuations in titanium alloys. Acta Mater..

[28] Wu D., Zhang L.G., Bai W.M., Zeng L.J. (2018). Effect of Fe content on microstructures and prop-erties of Ti6Al4V alloy with combinatorial approach. Trans. Nonferrous Met. Soc. China.

[29] Zeng L., Liu L., Huang S., Zhang L. (2017). Experimental investigation of phase equilibria in the Ti-Fe-Cr ternary system. Calphad.

[30] Wang J.L., Liu L.B., Zhang X.D., Bai W., Chen C.P. (2014). Isothermal Section of the Ti-Nb-Sn Ternary System at 700 °C. J. Phase Equilibria Diffus..

[31] Zeng L., Xu G., Liu L., Bai W., Zhang L. (2018). Experimental investigation of phase equilibria in the Ti-Fe-Zr system. Calphad.

[32] Wu D., Wang W.L., Zhang L.G., Wang Z.Y., Zhou K.C., Liu L.B. (2019). New high-strength Ti-Al-V-Mo alloy: From high-throughput composition design to mechanical properties. Int. J. Min. Met. Mater..

[33] Zhao J.-C. (2004). Reliability of the diffusion-multiple approach for phase diagram mapping. J. Mater. Sci..

[34] Zhao J.-C., Zheng X., Cahill D.G. (2005). High-throughput diffusion multiples. Mater. Today.

[35] Zheng Y., Williams R.E., Sosa J.M., Alam T., Wang Y., Banerjee R., Fraser H.L. (2016). The indirect influence of the ω phase on the degree of refinement of distributions of the α phase in metastable β-Titanium alloys. Acta Mater..

[36] Jones N.G., Dashwood R.J., Jackson M., Dye D. (2009). β Phase decomposition in Ti-5Al-5Mo-5V-3Cr. Acta Mater..

[37] Jones N.G., Dashwood R.J., Jackson M., Dye D. (2009). Development of chevron-shaped α precipitates in Ti-5Al-5Mo-5V-3Cr. Scr. Mater..

[38] Srinivasu G., Natraj Y., Bhattacharjee A., Nandy T.K., Nageswara Rao G.V.S. (2013). Tensile and fracture toughness of high strength β titanium alloy, Ti-10V-2Fe-3Al, as a function of rolling and solution treatment tem-peratures. Mater. Des..

[39] Fan J.K., Li J.S., Kou H.C., Hua K., Tang B. (2014). The interrelationship of fracture toughness and micro-structure in a new near β titanium alloy Ti-7Mo-3Nb-3Cr-3Al. Mater. Charact..

[40] Salvador C., Opini V.C., Mello M.G., Caram R. (2019). Effects of double-aging heat-treatments on the microstructure and mechanical behavior of an Nb-modified Ti-5553 alloy. Mater. Sci. Eng. A.

[41] Liang S., Yin L., Jiang R., Zhang X., Ma M., Liu R. (2014). Strengthening mechanism of two-phase titanium alloys with basketweave microstructure. J. Alloys Compd..

[42] Sun B.B., Sui M.L., Eckert J., Ma E., Wang Y.M., He G. (2006). Ultrafine composite microstructure in a bulk Ti alloy for high strength, strain hardening and tensile ductility. Acta Mater..

[43] Ma M., Wang D., Wu B. (1984). On the Law of Mixture in Dual Phase Steels. Mech. Behav. Mater..

[44] Cahoon J.R., Broughton W.H., Kutzak A.R. (1971). The determination of yield strength from hardness meas-urements. Metall. Trans..

[45] Zhang P., Li S., Zhang Z. (2011). General relationship between strength and hardness. Mater. Sci. Eng. A.

[46] Qin D.Y., Lu Y.F., Guo D., Zheng L., Liu Q., Zhou L. (2013). Tensile deformation and fracture of Ti-5Al-5V-5Mo-3Cr-1.5Zr-0.5Fe alloy at room temperature. Mater. Sci. Eng. A.

[47] Zhao Q., Chen Y., Xu Y., Torrens R., Bolzoni L., Yang F. (2021). Cost-affordable and qualified powder metallurgy metastable beta titanium alloy by designing short-process consolidation and processing. Mater. Des..

[48] Dan R.X., Li S., Cai B., Zhu W.W., Ren F.Z., Attallah M.M. (2021). A high strength and low modulus met-astable β Ti-12Mo-6Zr-2Fe alloy fabricated by laser powder bed fusion in-situ alloying. Addit. Manuf..

[49] Xu R.J., Liu B., Yan Z.Q., Chen F., Guo W.M., Liu Y. (2019). Low-cost and high-strength powder metallurgy Ti-Al-Mo-Fe alloy and its application. J. Mater. Sci..

[50] Oh J.M., Park C.H., Yeom J.H., Hong J.K., Kang N., Lee S.W. (2020). High strength and ductility in low-cost Ti-Al-Fe-Mn alloy exhibiting transformation-induced plasticity. Mater. Sci. Eng. A.

[51] Ren L., Xiao W.L., Kent D., Wan M., Ma C.L., Zhou L. (2020). Simultaneously enhanced strength and duc-tility in a metastable β-Ti alloy by stress-induced hierarchical twin structure. Scr. Mater..

[52] Zou Z., Simonelli M., Katrib J., Dimitrakis G., Hague R. (2021). Microstructure and tensile properties of additive manufactured Ti-6Al-4V with refined prior-β grain structure obtained by rapid heat treatment. Mater. Sci. Eng. A.

